# Dominant mutations in ITPR3 cause Charcot‐Marie‐Tooth disease

**DOI:** 10.1002/acn3.51190

**Published:** 2020-09-19

**Authors:** Julius Rönkkö, Svetlana Molchanova, Anya Revah‐Politi, Elaine M. Pereira, Mari Auranen, Jussi Toppila, Jouni Kvist, Anastasia Ludwig, Julika Neumann, Geert Bultynck, Stéphanie Humblet‐Baron, Adrian Liston, Anders Paetau, Claudio Rivera, Matthew B. Harms, Henna Tyynismaa, Emil Ylikallio

**Affiliations:** ^1^ Stem Cells and Metabolism Research Program Faculty of Medicine University of Helsinki Helsinki Finland; ^2^ Molecular and Integrative Biosciences Research Program Faculty of Bio‐ and Environmental Sciences University of Helsinki Helsinki Finland; ^3^ Institute for Genomic Medicine Columbia University Medical Center New York New York USA; ^4^ Precision Genomics Laboratory Columbia University Irving Medical Center New York New York USA; ^5^ Department of Pediatrics Columbia University Irving Medical Center New York New York USA; ^6^ Clinical Neurosciences Neurology University of Helsinki and Helsinki University Hospital Helsinki Finland; ^7^ Department of Clinical Neurophysiology Medical Imaging Center Helsinki University Central Hospital Helsinki Finland; ^8^ Neuroscience Center Helsinki Institute of Life Science University of Helsinki Helsinki Finland; ^9^ Department of Microbiology and Immunology Laboratory of Adaptive Immunity KU Leuven Leuven Belgium; ^10^ VIB‐KU Leuven Center for Brain and Disease Research Leuven Belgium; ^11^ Laboratory of Molecular and Cellular Signaling Department of Cellular and Molecular Medicine & Leuven Kanker Instituut KU Leuven Leuven Belgium; ^12^ Laboratory of Lymphocyte Signalling and Development Babraham Institute Cambridge United Kingdom; ^13^ Department of Pathology HUSLAB and University of Helsinki Helsinki Finland; ^14^ Institut de Neurobiologie de la Méditerranée INMED UMR901 Marseille France; ^15^ Department of Neurology Columbia University New York New York USA; ^16^ Department of Medical and Clinical Genetics University of Helsinki Helsinki Finland

## Abstract

**Objective:**

*ITPR3*, encoding inositol 1,4,5‐trisphosphate receptor type 3, was previously reported as a potential candidate disease gene for Charcot‐Marie‐Tooth neuropathy. Here, we present genetic and functional evidence that *ITPR3* is a Charcot‐Marie‐Tooth disease gene.

**Methods:**

Whole‐exome sequencing of four affected individuals in an autosomal dominant family and one individual who was the only affected individual in his family was used to identify disease‐causing variants. Skin fibroblasts from two individuals of the autosomal dominant family were analyzed functionally by western blotting, quantitative reverse transcription PCR, and Ca^2+^ imaging.

**Results:**

Affected individuals in the autosomal dominant family had onset of symmetrical neuropathy with demyelinating and secondary axonal features at around age 30, showing signs of gradual progression with severe distal leg weakness and hand involvement in the proband at age 64. Exome sequencing identified a heterozygous *ITPR3* p.Val615Met variant segregating with the disease. The individual who was the only affected in his family had disease onset at age 4 with demyelinating neuropathy. His condition was progressive, leading to severe muscle atrophy below knees and atrophy of proximal leg and hand muscles by age 16. Trio exome sequencing identified a *de novo ITPR3* variant p.Arg2524Cys. Altered Ca^2+^‐transients in p.Val615Met patient fibroblasts suggested that the variant has a dominant‐negative effect on inositol 1,4,5‐trisphosphate receptor type 3 function.

**Interpretation:**

Together with two previously identified variants, our report adds further evidence that *ITPR3* is a disease‐causing gene for CMT and indicates altered Ca^2+^ homeostasis in disease pathogenesis.

## INTRODUCTION

Charcot‐Marie‐Tooth disease (CMT) is a group of hereditary neuropathies, characterized by progressive distal sensory and motor impairment, which affects 1:2500 individuals.^1^ The disease is categorized into demyelinating CMT1, where median motor nerve conduction velocity (NCV) is < 38 m/s and axonal CMT2 where median motor NCV is> 38 m/s but compound muscle action potentials are (CMAP) decreased. Cases with features of both demyelination and axonopathy and NCV in the 30‐45 m/s range are sometimes referred to as intermediate CMT.^2^ A large number of CMT disease gene discoveries have led to insights of the disease mechanisms and potential therapies.^3^ Dominant variants in *ITPR3*, which encodes the inositol 1,4,5‐trisphosphate (IP_3_) receptor (IP_3_R) type 3, were recently suggested as potential causes of CMT.^4^, ^5^ Linkage analysis combined with exome sequencing revealed a p.Thr1424Met variant that segregated with a CMT phenotype in three patients from a single family.^4^ Furthermore, gene panel screening found a p.Met1064Val variant in a single index case for which no additional clinical details were provided.^5^ The pathogenicity of these variants was not confirmed by functional studies or by segregation of the variants in additional families.

Humans have three IP_3_R isoforms: IP_3_R1, IP_3_R2, and IP_3_R3. They are homologous in sequence but differ in physiological functions and tissue expression.^6^, ^7^ IP_3_ is produced after activation of G protein coupled receptors (GPCR), and binds the tetrameric IP_3_Rs, which release Ca^2+^ from ER into cytoplasm.^8^ The resulting elevation of intracellular Ca^2+^ concentration has several downstream effects on the cell.^9^ The importance of IP_3_R signaling in neurons is underscored by the defects in IP_3_R1 leading to ataxia or Gillespie syndrome.^10^, ^11^, ^12^, ^13^ Furthermore, the ER associated degradation pathway of activated IP_3_Rs is disrupted by inactivating variants in the genes *ERLIN1*, *ERLIN2,* and *RNF170*, causing hereditary spastic paraplegia and other neurodegenerative diseases.^14^, ^15^ IP_3_R3 itself has been implicated in apoptosis control, while alterations in its activity and/or expression levels drive oncogenesis and impact the survival of malignant cells.^16^, ^17^

In this study, we provide confirmatory evidence of the association of *ITPR3* with CMT. We introduce a CMT family with autosomal dominant mutation and one case with *de novo* mutation in *ITPR3*. In addition, we provide functional evidence of altered Ca^2+^ dynamics in patient fibroblasts.

## METHODS

### Patients and sequencing

Individuals P1, P2, P3, and P4 gave written informed consent and the ethics review board of HUS Helsinki University Hospital approved the study. Control fibroblast cells were from anonymous donors, who consented to use of the cells in scientific research. The fibroblast cells were collected from skin biopsy and cultured in Dulbecco’s Modified Eagle Medium (DMEM), supplemented with 10% FBS (Life Technologies), 1% penicillin/streptomycin (Life Technologies), 1% L‐glutamine (Life Technologies), and 0.2% uridine (Sigma). Cells were incubated at 37 *°C* in 5 % CO2.

Exome sequencing for the Finnish family was performed as described previously.^18^ Sanger sequencing primers are shown in Table S1. Research trio whole‐exome sequencing (WES) was done on individual P5 and parents after written informed consent was obtained through an institutional review board‐approved research study at the Institute for Genomic Medicine at Columbia University (protocol AAAO8410). DNA was extracted from maternal, paternal, and proband samples, exome sequenced on a HiSeq 2500 or NovaSeq 6000 with the Kapa Biosystem’s Library Preparation Kit, and whole‐exome captured with NimbleGen SeqCap EZ v.3.0 rapid or v.4.

### Western blot

Fibroblasts were lysed in RIPA buffer (Cell Signaling #9806) and an aliquot containing 10 µg of total protein was boiled, separated in 4‐20% Criterion™TGX™ gels, and transferred to 0.2 µm nitrocellulose using Trans‐Blot Turbo (all Bio‐Rad). Blocking was done with 10% milk in 0.1% TBS‐T. Antibodies were: IP_3_R1^(CT‐1)^, IP_3_R2^(NT‐2)^ (gift from Dr. David Yule, University of Rochester, NY), IP_3_R3 (BD‐Transduction #610312), and β‐tubulin (Cell Signaling #2146S). Anti‐rabbit and anti‐mouse (Jackson Immunoresearch#111‐035‐144 and #115‐035‐146) secondary antibodies were used. Detection was done by WesternBright™ ECL‐spray (Thermo Scientific) and imaging with Molecular Imager ChemiDoc XRS + with ImageLab (Bio‐Rad).

### Quantitative PCR and siRNA

RNA was extracted by NucleoSpin® RNA extraction kit (Macherey‐Nagel #740955) and reverse transcribed by Maxima first strand cDNA synthesis kit (Thermo Fischer). RT‐qPCR was performed using DyNAmo Flash SYBR Green qPCR Kit (Thermo Scientific) with specific primers for *ITPR1, ITPR2, ITPR3,* and *GAPDH* (Table S2) using Bio‐Rad CFX Maestro 1.1 software (Bio‐Rad). *ITPR3* siRNA knockdown experiments were conducted as described^19^ using *ITPR3* ON‐TARGETplus SMARTpool siRNA (Dharmacon #L‐006209‐00‐0005) and ON‐TARGETplus Non‐targeting siRNA (Dharmacon #D‐001810‐01‐05).

### Fibroblast Ca^2+^ imaging

We performed fibroblast Ca^2+^ imaging by two different methods in two different laboratories: non‐ratiometric manual Ca^2+^ assay using ATP stimulation (performed in University of Helsinki), and ratiometric automated Ca^2+^ assay using ionomycin, thapsigargin, and bradykinin stimulation (performed in KU Leuven).

### Non‐ratiometric manual Ca^2+^ assay

Cells were washed two times with Hank’s Balanced Salt Solution (HBSS) (in mM: 130 NaCl, 2.5 KCl, 1.8 CaCl_2_, 1.2 MgCl_2_, 10 HEPES, pH 7.4) and incubated for 50 min in dark in room temperature with 5 µg/ml Fluo‐4 AM Ca^2+^ indicator. After incubation, cells were washed three times with HBSS. For imaging, coverslips were placed on MatTek glass bottom dish (MatTek #P35G‐1.5‐14‐C) and imaged with Zeiss Axio Observer Z1 inverted phase contrast fluorescence microscope. During experiment, cells were perfused with Multichannel systems PPS2 Peristaltic perfusion system. Before every experiment, cells were allowed to rest for 5 min in the stage under HBSS perfusion. The excitation light was filtered through 494 nm band pass filter and the emission light passed through a 506 nm band pass filter. Emission wavelength was captured by Photometrics Prime BSI sCMOS Camera with ZEISS ZEN 3.1 (blue edition) imaging software. Acquisition protocol lasted a total of 12 minutes under constant perfusion at a rate of 2 ml/min. The cells were first perfused with HBSS containing 1.8 mM Ca^2+^ for 3 min and then with HBSS solution containing no Ca^2+^ (0‐Ca^2+^ HBSS) for another 3 min. Then Ca^2+^ release from ER was evoked by perfusion with 0‐Ca^2+^ HBSS containing 80 µmol/L adenosine 5’‐triphosphate (ATP) magnesium salt (Sigma‐Aldrich #A9187) for 2 min. After ATP‐evoked Ca^2+^ response, perfusion solution was changed back to 0‐Ca^2+^ HBSS for 2 min and finally back to 1.8 mmol/L Ca^2+^ containing HBSS solution for another 2 min to induce store‐operated Ca^2+^ entry (SOCE) as a positive control.

The results were analyzed using MatLab (MATLAB R2019b) and RStudio (version 1.2.5033). Regions of interests (cells) were masked from each experiment and the mean pixel intensity was measured at each time point (frame) using a modified version of the previously described MatLab script.^20^, ^21^ The baseline (F_0_) was selected from the first 0‐ Ca^2+^ period, from frames with stable intensity values. Relative intensities were calculated by first subtracting the baseline value from each frame and then dividing it by the baseline value [ΔF_t_/F_0_=(F_t_‐F_0_)/F_0_]. To analyze the kinetics of the Ca^2+^ response peaks, we created an R‐script, which allows automatic analysis of the peaks. The area under the curve (AUC), peak amplitude and time to peak were measured from each cell, and the averages per coverslip were calculated. The scripts used are available online (https://github.com/JuliusRonkko/Ca2‐analysis).

### Ratiometric automated Ca^2+^ assay

Cytosolic Ca^2+^ levels of fibroblasts seeded in 96‐well plates (Greiner) were monitored using ratiometric fluorescent Ca^2+^ indicator dye Fura‐2 AM (Eurogentec, Belgium). They were loaded with the Fura2‐AM (1 µmol/L) at RT for 30 min in a modified Krebs solution (in mM: 150 NaCl, 5.9 KCl, 1.2 MgCl_2_, 11.6 HEPES (pH 7.3), and 1.5 CaCl_2_). After loading, cells were rested for 30 min at RT in the absence of Fura‐2 AM to allow complete dye de‐esterification before proceeding to analysis on a FlexStation 3 microplate reader (Molecular Devices, Sunnyvale, CA, USA). The Ca^2+^ indicator was alternately excited at 340 and 380 nm and emission of fluorescence at 510 nm recorded. EGTA was added after 30 seconds in all conditions at a final concentration of 3 mmol/L. After another 60 seconds, cells were exposed to stimuli prepared in Ca^2+^‐free modified Krebs solution containing 3 mmol/L EGTA and Ca^2+^ transients were monitored for 6 min. Ionomycin and the irreversible SERCA‐inhibitor thapsigargin were both added at a final concentration of 10 µmol/L and bradykinin at a final concentration of 50 nmol/L. All traces are shown as the ratio of both emission wavelengths F_340_/F_380_ and were smoothened using a running average of 5. For quantification purposes, a baseline value was determined for each measurement as mean fluorescence between 30 sec and 90 sec. Fluorescence ratio F_340_/F_380_ was then normalized to the baseline values, and AUC of the peak, the peak amplitude (both analyzed between 90 and 450 sec) and the time to the peak were measured.

## RESULTS

### Autosomal dominant CMT family

**Figure 1:**
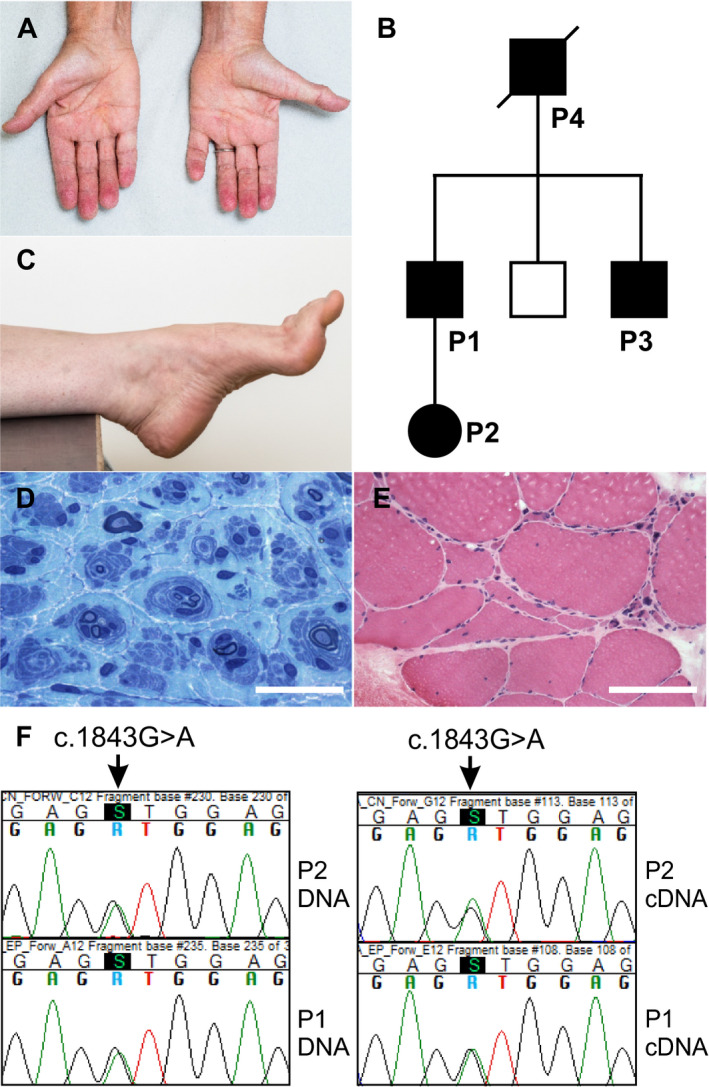
Clinical features and sequencing. Photographed at age 64, the index patient P1 had distal muscle atrophy (A). In the family, his father (P4) and one of his two brothers (P3) had been similarly affected (B). Moreover his daughter (P2) had had foot deformities since an early age and was noted to have pes cavus when examined at age 35 (C). In sural nerve biopsy of P1, a clear hypertrophic neuropathy with prominent onion bulbs was seen (D, plastic section toluidine blue staining, scale bar 25 µm). Muscle biopsy from tibialis anterior muscle of P1 showed prominent small group atrophy, fiber type grouping and secondary myopathic change (E, frozen section HE‐staining, scale bar 125 µm). Sanger sequencing confirmed the presence of *ITPR3* c.1843G> A (p.Val615Met) in all affected individuals, shown are P1 and P2 chromatograms (F), and it was absent in the unaffected brother.

The proband of the Finnish family (P1) first came to neurologic investigations at age 38. His first symptoms had started slightly before age 30 with weakened foot dorsiflexion and tendency to foot drop. Around age 33 he also started to experience increased clumsiness in his hands. He was last evaluated at age 64. His symptoms have been slowly progressive with increasing difficulty in walking in rough terrain. He uses supportive insoles but has remained ambulant without external aids. He has severe muscle atrophy in his lower legs, hammer toes, pes cavus, and thenar muscle atrophy (Fig. 1A). Sensation to light pressure was decreased distally from wrists and in foot soles. Vibration sense was decreased at ankles but present at wrists. Foot dorsiflexion or plantarflexion did not overcome gravity. Deep tendon reflexes were absent. His other diseases were hypothyroidism, hypercholesterolemia, and severe obstructive sleep apnea.

The proband had two brothers (Fig. 1B), one of whom had no neuropathic symptoms, while the other (P3) had pes cavus and progressive distal muscle weakness and wasting. His diseased father (P4) had been diagnosed with hereditary neuropathy of unknown cause but was otherwise relatively healthy and lived to an age of 93 years. He had remained ambulant until that age. The daughter of the proband (P2) first came to neurologic examination at age 35. She had had hammertoes and other deformities of the small bones of the feet since childhood, which had been operated on first at age 27. Despite this, she was ambulant without aids and able to play sports. She was able to walk on toes and heels. Marked pes cavus and hammertoes were noted (Fig. 1C), in addition to mild impairment of vibration sense at the right ankle.

Nerve conduction studies (NCS) of P1, P2, and P3 were consistent with demyelinating neuropathy, which were graded at least mild. The reduction in NCV was clearly less than is typical for CMT1A (Table 1). In addition, there was variable degrees of axonal neuropathy, which tended to worsen with age. Biopsies from P1 had been obtained at age 38. In sural nerve biopsy, a clear hypertrophic neuropathy with prominent onion bulbs was seen (Fig. 1D). Muscle biopsy from tibialis anterior muscle showed prominent small group atrophy, fiber type grouping, and secondary myopathic change (Fig. 1E).

**Table 1:** Clinical and neurophysiological findings.

Case	P2	P1	P3	P4	P5
Ethnicity	Finnish	Finnish	Finnish	Finnish	Ashkenazi Jewish
Sex	F	M	M	M	M
Age at onset	20s	27	n.a.	n.a.	4
Age at examination	35	63	31	Died age 93	16
Distal weakness	‐	+	+	+	+
Distal sensory impairment	+	+	+	+	+
Foot skeletal deformity	+	+	+	n.a.	+
Medianus motor conduction velocity m/s	39	33	45	n.a.	32
Medianus motor distal latency ms	4.92	7.48	4.1	n.a.	7.34
Medianus CMAP amplitude mV dist/prox	6.6/5.8	3.8/2.7	n.a.	n.a.	4.0/2.9
Radialis sensory conduction velocity m/s	52	48	n.a.	n.a.	n.a
Radialis SNAP amplitude µV	15	7.0	n.a	n.a.	n.a
Median and ulnar SNAP amplitude µV	n.a	n.a	n.a	n.a	6.7/5.1
Peroneus motor conduction velocity m/s	30	n.r.	43	n.a.	n.r
Peroneus motor distal latency ms	5.79	n.r.	6.4	n.a.	n.r
Peroneus CMAP amplitude dist/prox mV	4.9/3.8	n.r.	n.a.	n.a.	n.r
Tibialis CMAP amplitude dist mV	1.1	n.r.	n.a.	n.a.	n.r
Tibialis motor distal latency ms	4.76	n.r.	n.a	n.a.	n.r
Suralis sensory conduction velocity m/s	42	n.r.	35	n.a.	n.r
Suralis SNAP amplitude µV	1.8	n.r.	4.3	n.a.	n.r

CMAP: compound muscle action potential, SNAP: sensory nerve action potential, n.r. no response, n.a. not available.

### Single affected individual

The proband of the Ashkenazi Jewish family (P5) was developing normally until 4 years of age when he began falling. An evaluation at that time was concerning for pes cavus with hammertoes, and a motor only NCS showed a small tibial CMAP (0.5 mV) with a demyelinating range conduction velocity (20 m/sec). The median CMAP was normal (4.0 mV) but the NCV was 35 m/sec. No temporal dispersion or conduction block was present in either nerve. He was diagnosed with a demyelinating CMT and over the intervening decade he experienced progressive loss of leg strength and sensation in a symmetric distal to proximal gradient. After this evaluation he was not evaluated by a neurologist until 16 years of age when he established care at Columbia University Irving Medical Center (CUIMC).

On neurological examination at age 16, he had pes planus with complete loss of muscle below the knees, and atrophy of proximal leg and hand intrinsic muscles. There was no movement at the ankles, anti‐gravity strength at the knees, and mild weakness of hip flexion and the hand intrinsics. Temperature, pin prick, and vibration were severely reduced at the toes and ankles, but remarkably, joint position sense was preserved. All reflexes were absent. Otherwise, general examination, cognitive evaluation, and neurological examination were normal.

The proband was the product of a consanguineous union (parents are second cousins). His father is known to have flat feet and mildly reduced distal sensation in his early 40’s but no demonstrable weakness. His mother’s examination was normal. Of the proband’s 11 full siblings, none are suspected by the family to have similar symptoms but have not been formally tested.

Repeat EMG/NCS showed absent motor and sensory responses in the legs (Table 1). The median and ulnar nerves showed normal CMAP amplitudes and slowed conduction velocities (32 m/sec), with mildly reduced SNAP amplitudes and conduction velocities of 38 m/sec.

### Genetic findings

In P1, we first excluded *PMP22* duplication, *MFN2* and *GJB1* mutations. After this we performed exome sequencing on P1 and P3. We filtered the exome data for (1) shared nonsynonymous changes (excluding in frame insertions/deletions), (2) absence in SNP database (dbSNP), (3) prevalence ≤ 10^‐5^ in the Finnish subpopulation of gnomAD (v2.1.1), and (4) CADD score^22^ of 15 or more. The analysis left nine variants (Table S3) of which c.1843G>A (p.Val615Met) in exon 16 of *ITPR3* (NM_002224.4) was of interest. We confirmed the segregation of the variant with disease in the family by Sanger sequencing (Fig. 1F). The other identified variants had no previous studied function in Schwann cells or suggested association with CMT.

In P5, a chromosomal analysis and SNP microarray were within normal limits though the SNP microarray showed long contiguous runs of homozygosity consistent with the history of consanguinity. A CMT gene panel that included sequencing and deletion/duplication analysis of 42 genes identified single missense variants of uncertain significance in each of two autosomal recessive CMT genes (*IGHMBP2* and *NDRG1)* without other coding or copy number variants in those genes. A clinical exome was reported as negative. Trio sequence data were subsequently analyzed with an updated version of the Institute for Genomic Medicine’s established trio sequencing framework^23^ which identifies ‘‘qualifying’’ genotypes not observed in the parents, 12,044 control individuals from the Institute for Genomic Medicine, or two external databases of 6,503 and 60,706 control individuals provided by the National Heart, Lung, and Blood Institute (NHLBI) Exome Sequencing Project (ESP6500SI [March 2013 release]) and the Exome Aggregation Consortium (ExAC Browser v.0.3 [January 2015 release]), respectively. The analysis identified a *de novo* missense variant in exon 55 of the *ITPR3* gene (NM_002224.4, c.7570C>T, p.Arg2524Cys). The variant is absent from the gnomAD V2.1.1 database, has a CADD score of 32.

**Figure 2:**
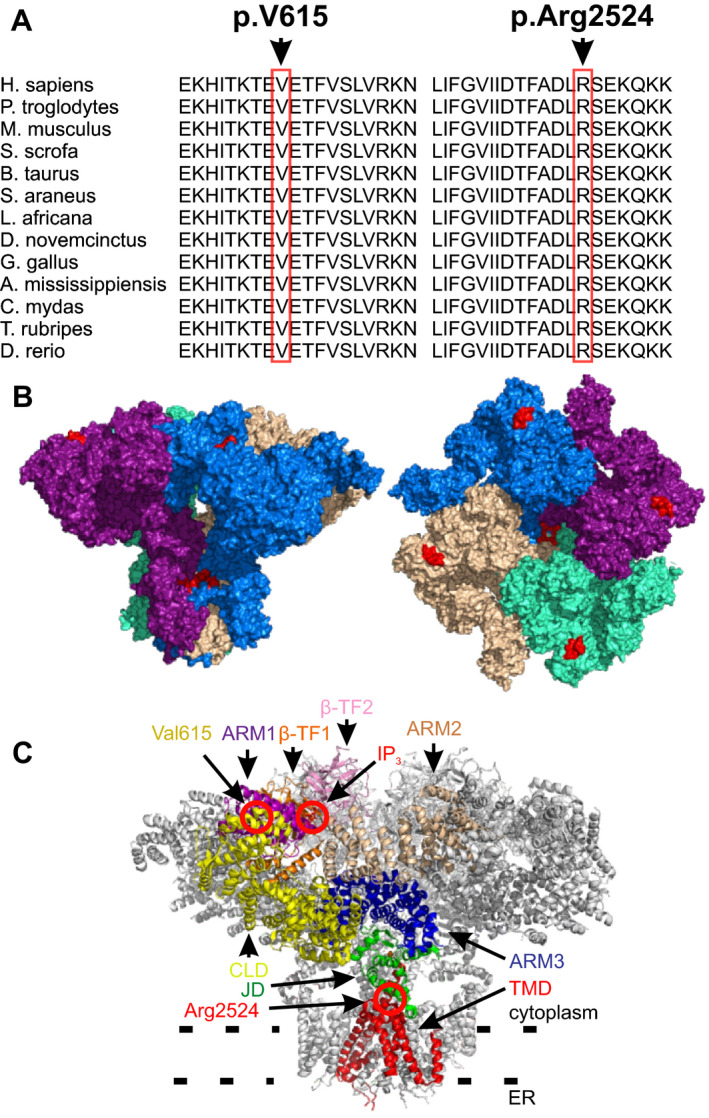
Position and conservation of IP_3_R3 mutations. The p.Val615Met and the p.Arg2524Cys mutations affect conserved stretch of amino acids (A). The p.Val615Met mutation lies adjacent to the cytoplasmic surface of IP_3_R3 and in proximity to the IP_3_ binding site, while the p.Arg2524Cys mutation lies in the channel pore (B‐D), as predicted based on the previously published model of the tetramer^23^. The amino acids 610‐620 and 2520‐2530 are highlighted in red (B and C). Key domains of IP_3_R3 channel and IP_3_‐molecule at its binding site are highlighted in the figure (D). ARM1‐3 = armadillo repeat domains 1‐3, BTF1‐2 = β‐trefoil domains 1‐2, CLD = center linker domain, JD = juxtamembrane domain, TMD = transmembrane domain.

Both *ITPR3* variants are predicted to be deleterious and damaging to protein structure and/or function based on in silico analyses (damaging by SIFT, probably damaging by PolyPhen2). The affected amino acids are highly conserved (Fig. 2A). We assessed the affected residues using published IP_3_R structures.^24^, ^25^, ^26^ p.Val615 is located in the armadillo repeat domain (ARM1), just after the IP_3_‐binding domain (running from aa 1 to ~ aa 600). p.Arg2524 is located in the transmembrane domain (TMD) and lies in the channel pore (Fig. 2B‐D).

### Protein and mRNA

Skin fibroblasts of P1 and P2 were available for study. We assessed the levels of IP_3_R proteins and corresponding mRNAs. IP_3_R3 protein level was decreased in the skin fibroblasts of P2 but not in P1, as compared with controls (Fig. 2E and F). However, P1 fibroblasts had significantly elevated *ITPR3* mRNA level as compared with controls (Fig. 2G), which suggests a compensatory mRNA upregulation to preserve the normal IP_3_R3 protein level. IP_3_R2 protein level was also decreased in P2, while those of IP_3_R1 were unaffected. Also, *ITPR1* and *ITPR2* mRNA levels were unchanged in both patient lines.

### Fibroblast Ca^2+^ flux

**Figure 3:**
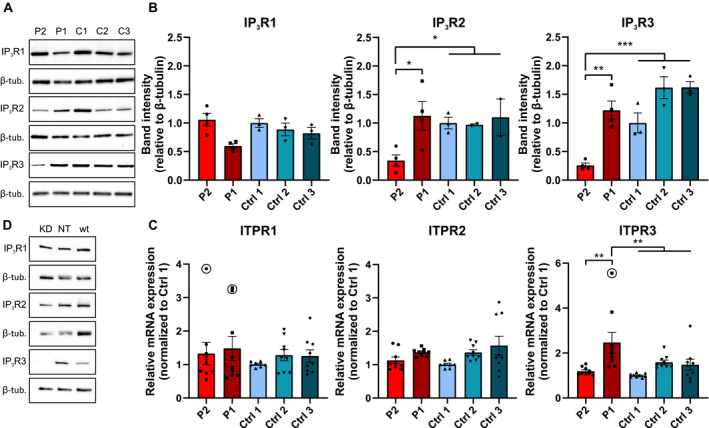
Fibroblast protein levels in Finnish family patients. Western blots of fibroblast lysates showed that the levels of IP_3_R1 were not changed between patient and control fibroblasts (A, B). In P2 fibroblasts, the levels of IP_3_R2 and IP_3_R3 were significantly decreased compared with the controls, whereas the levels in P1 fibroblasts were unchanged (A, B). qPCR showed that the mRNA level of *ITPR3* was increased in P1 but not in P2 fibroblasts (C). Finally, siRNA knockdown of *ITPR3* in control fibroblasts led to clear reduction in the protein level of IP_3_R3 by western blot, while the levels of IP_3_R1 and IP_3_R2 were unchanged (D) KD = *ITPR3* siRNA knockdown, NT = non‐targeting siRNA, wt = non‐treated. Data points marked in the figures were excluded from the data‐analysis (*P < 0.05, **P < 0.01, ***P < 0.001).

**Figure 4:**
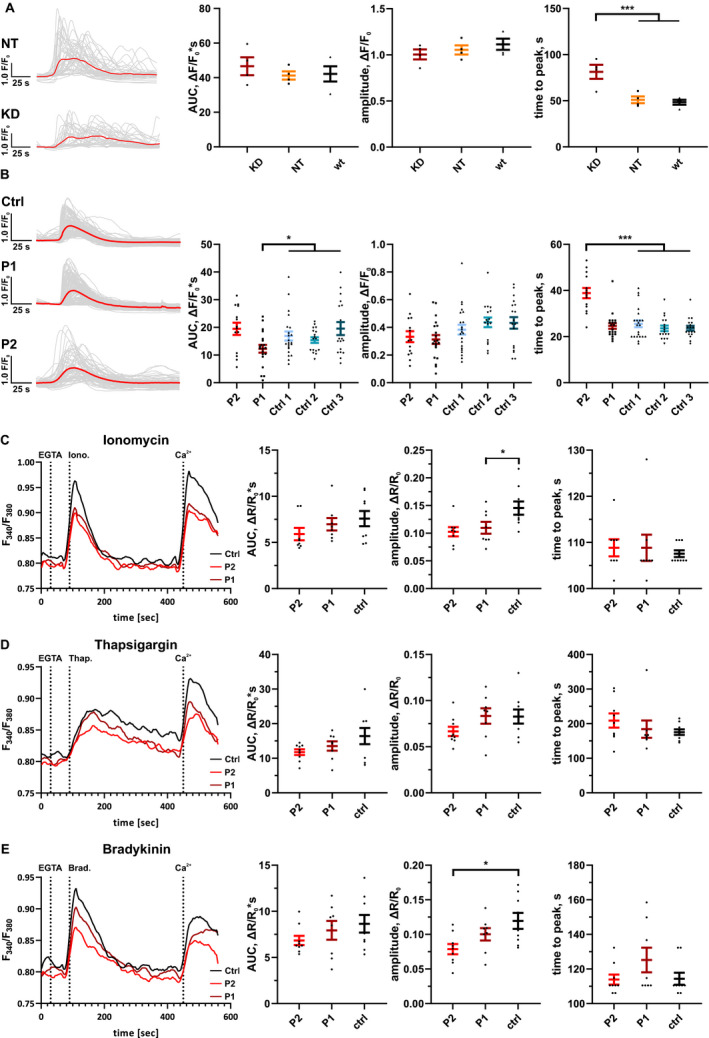
Ca^2+^ flux analysis in patient and control fibroblasts. The Ca^2+^ flux measurements were performed with two different methods, using cell‐permeant Fluo‐4 AM and Fura‐2 AM fluorescent Ca^2+^ indicators. In the first method (A and B), we used fluorescent microscopy to monitor Ca^2+^ in single cells, and 80 μmol/L ATP as GPCR agonist to evoke Ca^2+^ release. In each case, representative Ca^2+^ response curves after addition of ATP are shown. Grey thin lines are recordings from single cells whereas the thick line represents the averaged trace for all the cells in the given experiment. (A) siRNA of *ITPR3* led to increased average time to peak as compared with cells that were untreated (wt) or treated with non‐targeting (NT) siRNA (*n* = 4 individual experiments). KD = *ITPR3* siRNA knockdown, NT = non‐targeting siRNA, wt = non‐treated. (B) In response to ATP, P1 fibroblasts had decreased area under curve (AUC), while P2 cells had increased time to peak compared with unrelated controls (P1 *n* = 20, P2 *n* = 14 and three controls *n* = 17‐22 individual experiments). In the second method (C–E), we measured Ca^2+^ in single wells of a 96‐well plate, using 10 μmol/L ionomycin, 10 μmol/L thapsigargin or 50 nmol/L bradykinin to evoke responses, and compared patient cells to one control line performing five independent experiments in each setting. All stimuli (added after 90 sec, 2nd dotted line) were added in the presence of EGTA (added after 30 sec, 1st dotted line). (C) Traces showed a decrease in ionomycin‐induced Ca^2+^‐transients for both patients compared to the healthy control, with a significant decrease in peak amplitude for P2. (D) In response to SERCA inhibitor thapsigargin, patient fibroblasts did not display statistically significant decrease in Ca^2+^ ER store content compared to the healthy control. (E) In response to bradykinin, we observed a significant decrease in the peak amplitude of the response in P2 fibroblasts. All results are presented as mean ± SEM of independent experiments and statistical comparisons performed with one‐way ANOVA. (*P < 0.05, **P < 0.01, ***P < 0.001).

Next, we performed siRNA knockdown of *ITPR3* in control fibroblasts. The knockdown was confirmed by western blot (Fig. 3D) and led to altered Ca^2+^ flux dynamics in response to GPCR agonist ATP, with delayed peak of response but no change in amplitude or area under the curve (AUC) (Fig. 4A). This experiment confirmed that loss of IP_3_R3 produces a detectable phenotype in fibroblasts.

After this, we analyzed Ca^2+^ homeostasis in fibroblasts from P1 and P2 and healthy controls. The response to GPCR agonist ATP, which results in IP_3_ signaling and thus opening of IP_3_Rs, was analyzed by manual non‐ratiometric assay in the absence of extracellular Ca^2+^. The ATP‐evoked Ca^2+^ release was altered in both patient fibroblasts. P1 fibroblasts had statistically significant decrease in AUC, while P2 fibroblasts had increased time to peak (Fig. 4B). We performed additional Ca^2+^‐signaling analyses in cell populations using ratiometric automated technique. In these experiments, cells were first exposed to EGTA, an extracellular Ca^2+^ buffer. The response to the Ca^2+^ ionophore ionomycin, which provides an estimate of the total intracellular Ca^2+^ content, was decreased in P1 fibroblasts (Fig. 4C). The SERCA inhibitor thapsigargin, which gives an estimate of ER Ca^2+^ store content, did not cause statistically significant changes in the patient fibroblasts (Fig. 4D). Finally, the alternative GPCR agonist bradykinin evoked smaller cytosolic Ca^2+^ transients in P1 and P2 fibroblasts, the latter having significantly lower peak amplitude (Fig. 4E). Overall, the results suggest that the p.Val615Met mutation in *ITPR3* affects Ca^2+^ homeostasis and IP_3_‐mediated Ca^2+^ release.

## DISCUSSION

This study provides genetic and functional evidence for *ITPR3* as a dominant CMT disease gene. We describe two new mutations: p.Val615Met in adult onset and p.Arg2524Cys in childhood onset CMT. The reduction in median motor NCV in these patients was consistent with demyelinating neuropathy, which was also confirmed by nerve biopsy in one patient. However, the magnitude of reduction was less severe than is usually observed in CMT1A (OMIM #118220), which is the most common form of CMT.^2^ Our patients’ NCV was in the 30‐45 m/s range suggesting it should be considered in those with “intermediate CMT”.^2^ However, in CMT, conduction velocities vary by nerve, disease duration and patient, thus additional patients will be required to more conclusively define whether this is a demyelinating or intermediate CMT. Axonal involvement in our patients tended to become worse with age, which suggests that the axonal degeneration was secondary to demyelination.

Both of the identified mutations affect highly conserved residues. Being located in the central, modulatory region of IP_3_R3, shortly after the ligand‐binding domain,^24^ the p.Val615Met variant might influence IP_3_R3 activity, for example, through interfering with allosteric regulators.^9^ The p.Arg2524Cys variant, which localizes in the channel pore, may affect the channel properties and/or the ion flux directly, thus accounting for the earlier onset, faster progression, and more severe phenotype in this patient. The previously reported variant of unknown significance, p.Thr1424Met,^4^ was in a patient who similarly to our patients had moderately decreased median motor NCV of 34.7 m/s. Onset was at age 40, and two additional individuals in the same family were similarly affected.^4^ The p.Thr1424Met variant localizes in the armadillo repeat domain 2 near the subunit contact site. It may therefore affect oligomerization of the channel. Finally the variant p.Met1064Val, previously found in one index case of hereditary neuropathy,^5^ affects a conserved residue in the channel surface at the center linker domain. Similarly, as p.Val615Met and p.Arg2524Cys variants, both variants reported earlier are predicted to be deleterious and damaging to protein structure and/or function (damaging by SIFT, probably damaging by PolyPhen2). Thus the dominant missense variants may have different molecular effects on IP_3_R3 function, which also influences disease severity.

Our measurements of Ca^2+^ flux demonstrate altered Ca^2+^ homeostasis in p.Val615Met patient cells. The primary fibroblasts express *ITPR3* and thus are a useful tool to study the effects of the mutation under physiological conditions. The weakness of this model is that it does not account for possible neuron‐ or Schwann cell specific effects of the mutation. In addition, the results may be influenced by other genetic differences between control and patient cells in addition to the *ITPR3* mutation. Treatment with *ITPR3* siRNA was used to confirm that IP_3_R3 is active in fibroblasts under normal conditions and to model the effect of loss‐of‐function of the channel. We found altered GPCR agonist responses in both patient lines. The slowed response in P2 fibroblasts was similar but less pronounced than in siRNA‐treated cells. In addition, the P1 cells showed a decreased amplitude of the Ca^2+^ response to ATP. The differences in the Ca^2+^ responses between the two patient cell lines may be related to reduction in the amounts of IP_3_R2 and IP_3_R3 in P2 cells, which were compensated by *ITPR3* mRNA upregulation in P1 cells. In addition, the difference in sex or other possible genetic differences may in part account for the differences between P1 and P2 fibroblasts. Based on these results, the p.Val615Met variant may produce a dominant negative effect on channel function. The effect appears to be subtle, which is consistent with late onset and slowly progressive nature of our patients’ phenotype. As ionomycin and thapsigargin‐induced Ca^2+^ release tended to be lower in patient fibroblasts, it cannot be excluded that IP_3_R3 p.Val615Met is leaky, that is, has an increased likelihood of being open compared with the wild type situation, thereby lowering steady state ER Ca^2+^ levels and thus dampening Ca^2+^ release in the cytosol upon agonist exposure.

Our results suggest an important role of IP_3_R3 in peripheral nerve maintenance. This is supported by its localization in paranodal regions of rat Schwann cells, where its proximity to another CMT gene product, gap junction protein beta‐1 (GJB1), may allow swift propagation of Ca^2+^ signals from cell to cell.^27^ Abnormal Ca^2+^ flux could contribute to altered axonal Ca^2+^ microdomains that disturb mitochondrial transport, as has been suggested for dominant mutations in the plasma membrane cation channel transient receptor potential cation channel, subfamily V, member 4 (TRPV4)^28^, ^29^, ^30^, ^31^ which cause CMT2C^32^, ^33^, ^34^. Furthermore, mutations in other components of the IP_3_ signaling pathway, for example, *FIG4* and *SBF2*, cause demyelinating CMT,^35^, ^36^ which highlights the importance of this pathway for peripheral myelin maintenance. Finally, IP_3_R3 has important implications for regulation of mitochondrial function and cell death and survival by participating in Ca^2+^ transfer between ER and mitochondria,^16^, ^37^ a process which is also dependent on another important CMT gene, mitofusin 2 (*MFN2*).^38^ IP_3_R3 defects may decrease mitochondrial Ca^2+^ and predispose to defective bioenergetic or ER membrane function, which have been found in other forms of CMT.^39^, ^40^

In conclusion, our results provide further evidence that *ITPR3* is a disease gene for CMT. Additional studies, ideally in neuronal or animal models, will be needed to elucidate the effects of the disease variants on IP_3_R3 function and evaluate the potential of targeting Ca^2+^ flux as a therapeutic target in CMT.

## Author Contributions

J.R., S.M., M.A., G.B., S.H‐B., Ad.L., C.R., H.T., and E.Y. contributed to the conception and design of the study. J.R., S.M., M.A., J.T., J.N., A.P., and E.Y performed the experiments. J.R., A.R.P., E.M.P., J.K., An.L., J.N., G.B., and M.B.H contributed to the acquisition and analysis of data. J.R. and E.Y. wrote the manuscript with input from all authors.

## Conflicts of Interest

The authors report no competing interests.

## Supporting information


**Table S1**. Sequencing primers used for Sanger sequencing *ITPR3* DNA and cDNA.
**Table S2**. Primers used for quantitative reverse transcription PCR of *ITPR1*, *ITPR2*, *ITPR3*, and *GAPDH*.
**Table S3**. Filtering of exome sequencing data of P1 and P3 left nine variants. The variants were analyzed further *in silico*. The variants not found in gnomAD were Sanger sequenced in all family members. The analysis left *ITPR3* as a gene of interest.
