# Supplementary material for: Dominant mutations in ITPR3 cause Charcot‐Marie‐Tooth disease
Source: Ann Clin Transl Neurol. 2020 Sep 19;7(10):1962–72. doi: 10.1002/acn3.51190 (PMC7545616; doi:10.1002/acn3.51190)
Supplement: Supplementary file 1 — Table S1. Sequencing primers used for Sanger sequencing ITPR3 DNA and cDNA. Table S2. Primers used for quantitative reverse transcription PCR of ITPR1, ITPR2, ITPR3, and GAPDH. Table S3. Filtering of exome sequencing data of P1 and P3 left nine variants. The variants were analyzed further in silico. The variants not found in gnomAD were Sanger sequenced in all family members. The analysis left ITPR3 as a gene of interest. [file ACN3-7-1962-s001.docx]

**Supplementary table 1.** Sequencing primers.

| genomic DNA | Forward | *ITPR3*-ex16 | TTTCCTGGACCTC TGCCTTT |
| --- | --- | --- | --- |
| genomic DNA | Reverse | *ITPR3*-ex16 | GTTCACACAACCAGCAGAGT |
| cDNA | Forward | *ITPR3*-cDNA ex16 | GCAAGAACCAGGAGCACATT |
| cDNA | Reverse | *ITPR3*-cDNA ex16 | CACACACAGGTCAGAGAGGT |

**Supplementary table 2**. Quantitative PCR primers.

| **Gene** | **Primer** | **Sequence** |
| --- | --- | --- |
| *ITPR1* | Forward | CTCAACAAACTGCACCACGC |
| *ITPR1* | Reverse | AGGAGCTGGATCACATTGCC |
| *ITPR2* | Forward | TTCATCATGACCCATGCCGT |
| *ITPR2* | Reverse | TCAGGATTAAGCTCTGCAGCTA |
| *ITPR3* | Forward | CTTCATCAGCACTTTGGGGC |
| *ITPR3* | Reverse | ACCTTGAAGAGGCAGTCACG |
| *GAPDH* | Forward | CGCTCTCTGCTCCTCCTGTT |
| *GAPDH* | Reverse | CCATGGTGTCTGAGCGATGT |

**Supplementary table 3. Exome sequencing findings**

| Chr | pos | Ref | Alt | Gene | Most Severe Canonical Transcript Consequence | HGVS protein level | HGVS DNA level | OMIM | CADD C-score | gnomAD | Segregates with the phenotype |
| --- | --- | --- | --- | --- | --- | --- | --- | --- | --- | --- | --- |
| 1 | 19422101 | G | A | UBR4 | missense_variant | ENSP00000364403.3:p.Arg4522Trp | ENST00000375254.3:c.13564C>T |  | 35,00 |  | n.t |
| 1 | 19430739 | G | A | UBR4 | missense_variant | ENSP00000364403.3:p.Ser4247Phe | ENST00000375254.3:c.12740C>T |  | 25,30 |  | Yes |
| 1 | 32827403 | G | T | TSSK3 | intron_variant |  | ENST00000574315.1:c.-80-1793G>T |  | 23,20 |  | n.t |
| 1 | 32827403 | G | T | FAM229A | missense_variant | ENSP00000454338.1:p.Ser8Tyr | ENST00000428500.1:c.23C>A |  | 23,20 |  | n.t |
| 1 | 75198984 | A | G | CRYZ | 5_prime_UTR_variant |  | ENST00000417775.1:c.-397T>C |  | 33,00 | 8,24E-06 | n.t |
| 1 | 75198984 | A | G | TYW3 | missense_variant | ENSP00000359904.3:p.Asp19Gly | ENST00000370867.3:c.56A>G |  | 33,00 | 8,24E-06 | n.t |
| 6 | 33635698 | G | A | ITPR3 | missense_variant | ENSP00000363435.4:p.Val615Met | ENST00000374316.5:c.1843G>A | {Diabetes, type 1, susceptibility to}, 222100 (2), Autosomal recessive | 28,60 |  | Yes |
| 14 | 61190125 | C | T | SIX4 | missense_variant | ENSP00000216513.4:p.Gly223Asp | ENST00000216513.4:c.668G>A |  | 27,10 |  | Yes |
| 19 | 8660650 | T | C | ADAMTS10 | missense_variant | ENSP00000270328.4:p.Tyr492Cys | ENST00000270328.4:c.1475A>G | Weill-Marchesani syndrome 1, recessive, 277600 (3), Autosomal recessive | 24,20 |  | No |
| 19 | 15563475 | C | A | RASAL3 | missense_variant | ENSP00000341905.5:p.Arg941Leu | ENST00000343625.7:c.2822G>T |  | 17,58 | 0,0001 | n.t |
| 20 | 1317279 | G | A | AL136531.1 | stop_gained | ENSP00000440123.1:p.Arg18Ter | ENST00000537552.1:c.52C>T |  | 23,30 | 0,002085 | n.t |
| 20 | 1317279 | G | A | SDCBP2-AS1 | intron_variant |  | ENST00000609470.1:n.120+11106G>A |  | 23,30 | 0,002085 | n.t |

HGVS=human genome variant society, OMIM=online mendelian inheritance in man, CADD=combined annotation dependent depletion, gnomAD=genome aggregation database, n.t=not tested. Chromosome positions are from hg19.
